# *Exonuclease 1* genetic variant is associated with clinical outcomes of pemetrexed chemotherapy in lung adenocarcinoma

**DOI:** 10.7150/jca.78498

**Published:** 2022-12-07

**Authors:** Mi Jeong Hong, Ji Eun Park, Shin Yup Lee, Jang Hyuck Lee, Jin Eun Choi, Hyo-Gyoung Kang, Sook Kyung Do, Ji Yun Jeong, Kyung Min Shin, Won Ki Lee, Yangki Seok, Sun Ha Choi, Yong Hoon Lee, Hyewon Seo, Seung Soo Yoo, Jaehee Lee, Seung Ick Cha, Chang Ho Kim, Jae Yong Park

**Affiliations:** 1 Department of Biochemistry, School of Medicine, Kyungpook National University, Daegu, Republic of Korea.; 2 Cell and Matrix Research Institute, School of Medicine, Kyungpook National University, Daegu, Korea.; 3 Department of Internal Medicine, School of Medicine, Kyungpook National University, Daegu, Republic of Korea.; 4 BK21 Plus KNU Biomedical Convergence Program, Department of Biomedical Science, Kyungpook National University, Daegu, Korea.; 5 Department of Pathology, School of Medicine, Kyungpook National University, Daegu, Republic of Korea.; 6 Department of Radiology School of Medicine, Kyungpook National University, Daegu, Republic of Korea.; 7 Medical Research Collaboration Center in Kyungpook National University Hospital and School of Medicine, Kyungpook National University, Daegu, Republic of Korea.; 8 Department of Thoracic Surgery, Soonchunhyang University Gumi Hospital, Gumi, Korea.

**Keywords:** lung adenocarcinoma, miRNA target sites, genetic variants, chemotherapy, response, survival

## Abstract

Pemetrexed is an anti-folate agent which is one of the most frequently used chemotherapy agents for non-squamous non-small cell lung cancer (NSCLC) patients. However, clinical response to pemetrexed chemotherapy and survival outcome of patients varies significantly. We evaluated whether the genetic variants in miRNA target sites may affect the treatment outcome of pemetrexed chemotherapy in lung adenocarcinoma patients. One hundred SNPs in miRNA binding regions in cancer-related genes were obtained from the crosslinking, ligation, and sequencing of hybrids (CLASH) and CancerGenes database, and the associations with the response to pemetrexed chemotherapy and survival outcomes were investigated in 314 lung adenocarcinoma patients. Two polymorphisms, *EXO1* rs1047840G>A and *CAMKK2* rs1653586G>T, were significantly associated with worse chemotherapy response (adjusted odds ratio [aOR] = 0.41, 95% CI = 0.24-0.68, *P* = 0.001, under dominant model; and aOR = 0.33, 95% CI = 0.16-0.67, *P* = 0.002, under dominant model, respectively) and worse OS (adjusted hazard ratio [aHR] = 1.34, 95% CI = 1.01-1.77, *P* = 0.04, under dominant model; and aHR = 1.50, 95% CI = 1.06-2.13, *P* = 0.02, under dominant model, respectively) in multivariate analyses. Significantly increased luciferase activity was noted in *EXO1* rs1047840 A allele compared to G allele. In conclusion, two SNPs in miRNA binding sites, especially *EXO1* rs1047840G>A, were associated with the chemotherapy response and survival outcome in lung adenocarcinoma patients treated with pemetrexed.

## Introduction

A huge effort has been made to improve the prognosis of lung cancer patients over the past decades, and the treatment of lung cancer has progressed remarkably by recent innovations of targeted therapy and immunotherapy 1-3. However, cytotoxic chemotherapy alone or in combination with other cancer therapies remains as an important treatment modality for non-small cell lung cancer (NSCLC) patients. Pemetrexed is one of the most frequently used chemotherapeutic agents for non-squamous NSCLC patients. Recently, immune checkpoint blockade combined with pemetrexed and platinum doublet chemotherapy has been approved as a standard first-line treatment in patients without driver mutations, which significantly increased overall survival (OS) and progression-free survival (PFS) compared to previous standard of care chemotherapy in non-squamous NSCLC patients 4.

Pemetrexed is an anti-folate agent that inhibits cell replication and growth through interrupting synthesis of DNA and RNA by targeting three enzymes involved in purine and pyrimidine synthesis: thymidylate synthase (TS), dihydrofolate reductase (DHFR), and glycinamide ribonucleotide formyltransferase (GARFT) 5. It is widely accepted that the efficacy of pemetrexed is worse in squamous NSCLC and small-cell lung cancer than non-squamous NSCLC 6, but therapeutic outcomes of pemetrexed varies significantly even in patients with adenocarcinoma. Despite much effort to discover and validate molecular biomarkers to predict efficacy of pemetrexed, no viable biomarkers have yet been clearly established in clinical practice 7-9.

MicroRNA (miRNA) is a small (containing about 22 nucleotides) single-stranded non-coding RNA molecule that functions as endogenous negative regulators by binding to the 3'-untranslated region (UTR) of target mRNA causing degradation or translational inhibition of the target 10, 11. Comprehensive miRNA profiling demonstrated that miRNAs are involved in vital biological processes such as cell division and death, cellular metabolism, intracellular signaling, and immunity 12-15. In addition, miRNAs are critically involved in the development and progression of diverse human cancers 16, 17. Computational approach for miRNA target prediction have focused on canonical (seed-based) target sites, demonstrating that single nucleotide polymorphisms (SNPs) in miRNA binding sites could be used as cancer biomarkers for predicting cancer risk, therapeutic response and prognosis of patients 18. However, recent advances in transcriptome-wide mapping of miRNA target sites by crosslinking, ligation, and sequencing of hybrid (CLASH), elucidated that considerable portion of miRNA-mRNA interactions are mediated not only through canonical seed sites, but also through non-canonical sites 19. Based on the association between miRNA dysregulation and human malignancies, we hypothesized that SNPs in miRNA target sites may alter miRNA-mRNA binding and consequently gene expression, thus affecting the treatment response and survival outcomes of patients treated with pemetrexed. To test this hypothesis, we selected SNPs in miRNA target sites using CLASH data and analyzed the association with the clinical outcome of pemetrexed treatment in lung adenocarcinoma patients.

## Materials and Methods

### Study populations

A total of 314 lung adenocarcinoma patients with available genomic DNA samples who underwent pemetrexed based chemotherapy at Kyungpook National University Hospital (KNUH) in Daegu, Korea, between March 2007 and July 2015, were enrolled. We excluded patients who had any active malignancies ≤ 3 years before the diagnosis of lung cancer, except resected non-melanoma skin cancer and *in situ* cancers including carcinoma *in situ* of the cervix or breast. Patients who were diagnosed with stage III/IV or had recurred disease after curative surgery were treated with either pemetrexed plus cisplatin for up to 4 to 6 cycles as first line chemotherapy regimen with/without pemetrexed maintenance therapy, or pemetrexed alone as second or further line chemotherapy. Because pemetrexed maintenance therapy was approved in 2009 in Korea, patients who were treated with pemetrexed plus cisplatin before 2009 did not underwent pemetrexed maintenance therapy even if their disease had not progressed after four cycles of first-line pemetrexed/cisplatin chemotherapy 20. Chemotherapy was discontinued upon disease progression, major toxicities, or according to patient's or physician's decision. Response to chemotherapy was assessed according to the Response Evaluation Criteria in Solid Tumors 21 and the best overall response for each patient was reported. Patients with complete response or partial response were defined as responders, and patients with stable disease or progressive disease were defined as nonresponders. Survival outcome was assessed with overall survival (OS), the time between the first date of chemotherapy and date of death or last follow-up. Genomic DNA samples were provided by the National Biobank of Korea, KNUH, which is supported by the Ministry of Health, Welfare and Family Affairs. Written informed consent was obtained from all patients and this study was approved by the Institutional Review Board of the KNUH.

### SNP selection and genotyping

PolymiRTS database 3.0 (http://compbio.uthsc.edu/miRSNP) 22 was used to find potentially functional variants in miRNA binding region, and 24,027 SNPs were extracted from the CLASH data which has been integrated in PolymiRTS database. Out of these, 1,574 SNPs in genes involved in human cancer were selected using the CancerGenes database (http://cbio.mskcc.org/cancergenes) 23. Lastly, 100 SNPs were collected after excluding those with minor allele frequency < 0.05 in the HapMap-JPT and those in strong linkage disequilibrium (LD, *r*^2^ ≥ 0.8). Genotyping was performed using the iPLEX^®^ Assay and MassARRAY^®^ System (Agena Bioscience, San Diego, CA, USA).

### Cloning of the luciferase reporter gene and dual luciferase assay

Luciferase reporter assay was conducted to determine whether *EXO1* rs1047840G>A and *CAMKK2* rs1653586G>T modulate the binding of miRNA and thus affects the expression level of target genes. Luciferase reporter plasmids was constructed using the psiCHECK^TM^-2 vector (Promega, Madison, WI, USA). *EXO1* coding sequence containing rs1047840G or rs1047840A and *CAMKK2* 3'UTR sequence containing rs1653586G or rs1653586T were synthesized using PCR from human genomic DNA and cloned into the psiCHECK^TM^-2 vector. In the PolymiRTS database, we found that miR-30e and miR-185 were experimentally demonstrated to interact with the target sites on mRNAs of EXO1 and CAMKK2, respectively. Each construct was then co-transfected with miRNA (psiCHECK^TM^-2-*EXO1* with miR-30e, and psiCHECK^TM^-2-*CAMKK2* with miR-185) into H1299 cells following the manufacturer's instructions. After 24 hours of incubation, *Renilla* luciferase activities were measured using the firefly luciferase activities as a normalization control.

### Statistical analysis

Deviations from Hardy-Weinberg equilibrium was measured by a goodness-of-fit χ^2^ test. Genotypes for SNPs were analyzed as three-group categorical variable, and analyzed under dominant, recessive, and codominant models. The association between clinical variables or genotypes and response to chemotherapy was tested by odds ratio (OR) and 95% confidence interval (CI) using unconditional logistic regression. Kaplan-Meier method was used to estimate survival analysis, and log-rank test was used to compare the difference in OS according to different clinical variables or genotypes. Hazard ratio (HR) and 95% CI were estimated using multivariate Cox proportional hazards model. Variables with *P* value < 0.05 were considered statistically significant. The statistical data were obtained using Statistical Analysis System for Windows, version 9.4 (SAS Institute, Cary, NC, USA).

## Results

Associations between clinical parameters and the response to chemotherapy and survival outcomes are presented in Table 1. Overall response rate was 41.7% and median survival time was 24.1 months (95% CI = 21.7-25.9 months). Univariate analyses showed that age > 65, no benefit from tyrosine kinase inhibitor (TKI) treatment which include wild-type EGFR or ALK or no response to EGFR- or ALK-TKIs, pemetrexed maintenance therapy, and pemetrexed/cisplatin regimen were associated with better response. Regarding the OS, female sex, never-smoking status, postsurgical recurrence versus stage III/IV, ECOG performance status 0, benefit from TKI, pemetrexed maintenance therapy, and pemetrexed alone were significantly associated with the better OS. The conflicting results may be partly explained by first-line pemetrexed/cisplatin use in patients with wild-type EGFR or ALK and second-line pemetrexed monotherapy in patients with upfront EGFR- or ALK-TKI treatment, because the pemetrexed/cisplatin provides higher response rate than pemetrexed monotherapy but EGFR- or ALK- TKI is associated with better survival than chemotherapy alone.

**Figure 1:**
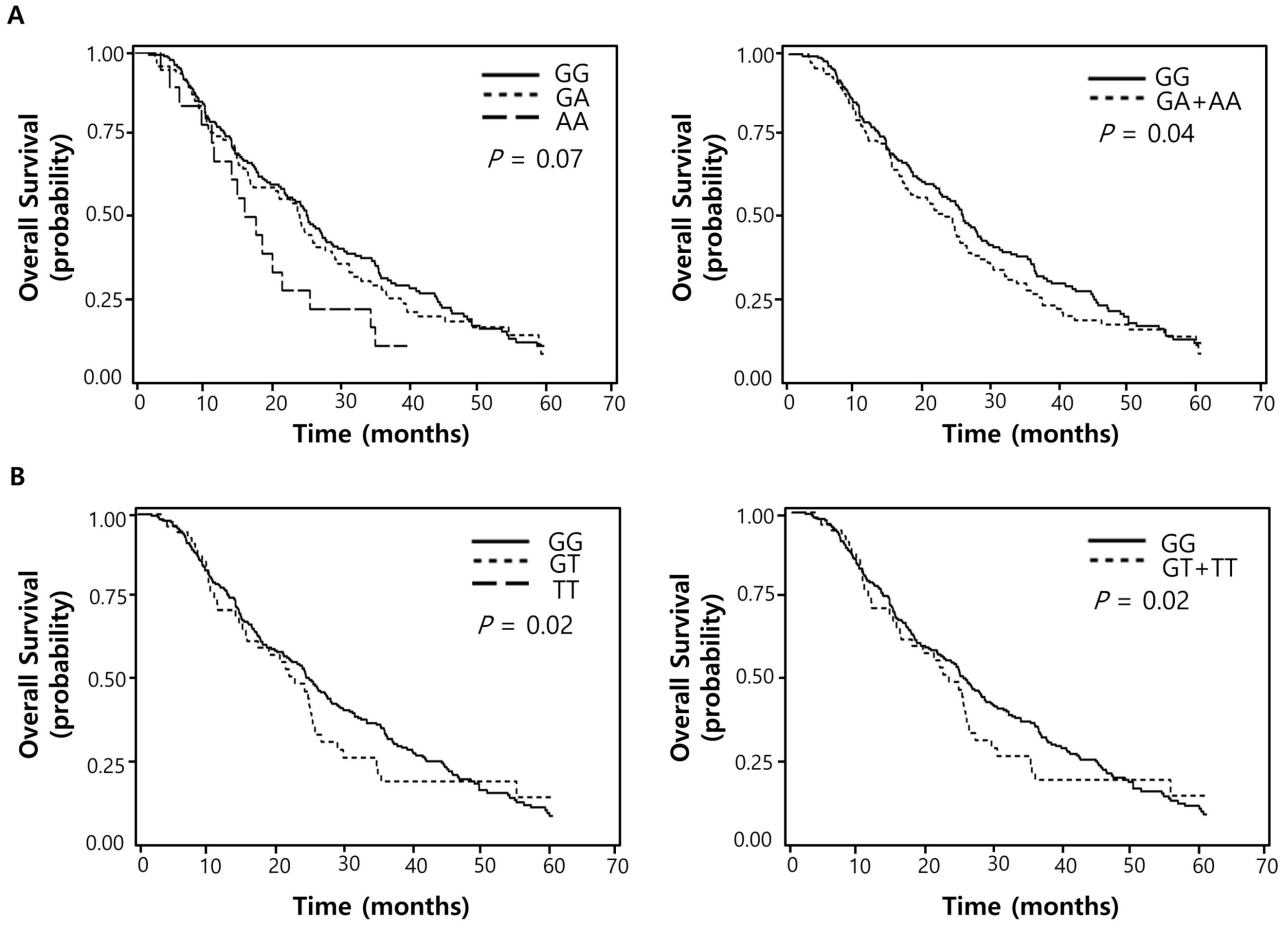
Overall survival curves according to (A) *EXO1* rs1047840G>A (B) *CAMKK2* rs1653586G>T genotypes. *P* values by multivariate Cox proportional hazard model.

Of the 100 SNPs genotyped, 74 SNPs were further analyzed (Supplementary Table 1) after excluding 3 SNPs with genotyping failure and 23 SNPs with deviations from Hardy-Weinberg equilibrium (*P* < 0.05) or low call rates (< 95%). Among these, 7 SNPs were significantly associated with the response to pemetrexed (Table 2) while 16 SNPs with OS (Table 3). Two polymorphisms (*EXO1* rs1047840G>A and *CAMKK2* rs1653586G>T) were significantly associated with both chemotherapy response and survival outcome. The *EXO1* rs1047840G>A and *CAMKK2* rs1653586G>T were significantly associated with worse chemotherapy response (adjusted odds ratio [aOR] = 0.41, 95% CI = 0.24-0.68, *P* = 0.001, under a dominant model; and aOR = 0.33, 95% CI = 0.16-0.67, *P* = 0.002, under a dominant model, respectively) and worse OS (adjusted hazard ratio [aHR] = 1.34, 95% CI = 1.01-1.77, *P* = 0.04, under a dominant model; and aHR = 1.50, 95% CI = 1.06-2.13, *P* = 0.02, under a dominant model, respectively) in multivariate analyses (Table 4 and Figure 1).

**Figure 2:**
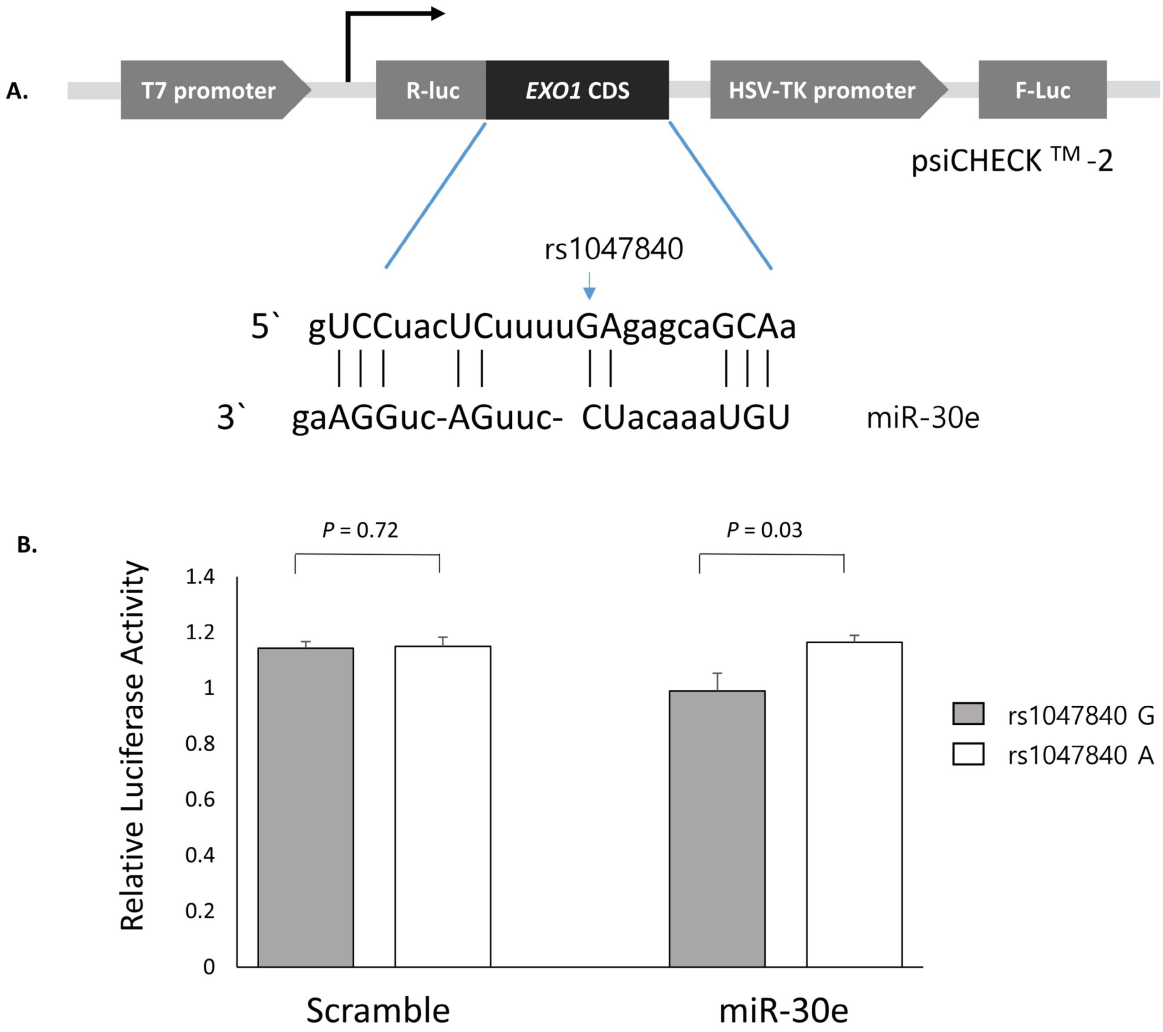
** Functional analysis of *EXO1* rs1047840G>A by dual luciferase reporter assay. (A)** Schematic representation of reporter plasmids containing *EXO1* rs1047840G>A, and the complementarity between miR-30e and *EXO1* coding sequence targeted. **(B)** *Renilla* luciferase assay for the effect of miRNA binding on rs1047840G>A using H1299 cells. *Renilla* luciferase activity was normalized to firefly luciferase activity and data are presented relative to the Mock control. Each bar represents mean ± SE. *P* values by Student's t-test. R-luc, *Renilla* luciferase; F-luc, firefly luciferase; HSV-TK, Herpes simplex virus thymidine kinase.

Next, to investigate whether the *EXO1* rs1047840G>A and *CAMKK2* rs1653586G>T modulates the binding of miRNAs and consequently influence the expression level of *EXO1* and *CAMKK2*, psiCHECK^TM^-2-*EXO1* containing rs1047840G>A and psiCHECK^TM^-2-*CAMKK2* containing rs1653586G>T were generated and co-transfected into H1299 cells with miR-30e and miR-185, respectively. The CLASH data showed that the binding between *EXO1* and miR-30e was non-canonical (Figure 2A) and luciferase assay showed that the *Renilla* luciferase activity was significantly increased in *EXO1* rs1047840A allele compared with rs1047840G allele (*P* = 0.03, Figure 2B). These results suggest that rs1047840G>A altered the binding of miR-30e and consequently increased the expression of *EXO1*. However, there was no difference in the luciferase activity between *CAMKK2* rs1653586 G and T alleles (data not shown).

## Discussion

In this study, we investigated whether the treatment outcomes of pemetrexed chemotherapy in lung adenocarcinoma patients are different according to the genotypes of genetic polymorphisms in miRNA target sites. The study revealed that two SNPs in miRNA binding sites, *EXO1* rs1047840G>A and *CAMKK2* rs1653586G>T, could predict the response to pemetrexed chemotherapy and survival outcome. The luciferase assay suggested that rs1047840G>A alters the binding of miRNA and consequently increases the expression level of *EXO1*. These findings suggest that the two SNPs, especially *EXO1* rs1047840G>A, could be used as potential biomarkers to predict therapeutic outcomes in lung adenocarcinoma patients treated with pemetrexed chemotherapy, which may help to establish an optimal personalized treatment strategy.

Human exonuclease 1 (EXO1) is a member of the Rad2/XPG family of nucleases which exhibits 5'→3' exonuclease, 5' flap endonuclease, and 5'→3' RNase H activity 24, 25. This enzyme mainly contributes to the regulation of cell cycle checkpoints and post-replicative DNA repair pathways, such as mismatch repair (MMR), translesion DNA synthesis (TLS), nucleotide excision repair (NER), and double-strand break repair (DSBR) 26-30. Dysfunction of *EXO1* could impair DNA repair processes which can cause replication stress followed by genomic instability and development of cancer 31. *EXO1* genetic variants have been associated with the risk and prognosis of different types of cancers, including lung cancer 32-37. In addition, *EXO1* overexpression and its relation to the resistance to chemotherapy or radiotherapy and poor prognosis was reported in multiple types of cancers 38-41. Pemetrexed interferes with folate metabolism leading to ineffective DNA synthesis and tumor cell growth failure 5. Inhibition of TS by pemetrexed induces uracil misincorporation into DNA resulting in cell death, and base excision repair (BER) system is responsible for the removal of misincorporated uracil. Therefore, the upregulation of BER is associated with pemetrexed resistance and is found in small cell and squamous cell carcinoma which are known as the pemetrexed-resistant histological subtypes of lung cancer 42. Likewise, given its role in DNA damage response and cell cycle checkpoint activation, the upregulation of *EXO1* may lead to the resistance to pemetrexed chemotherapy. In this study, *EXO1* rs1047840G>A was associated with worse clinical outcomes in lung adenocarcinoma patients after pemetrexed chemotherapy and linked to increased expression of *EXO1*. Based on our results, it is postulated that the rs1047840-induced change in miRNA binding efficiency may increase the expression level of *EXO1*, resulting in worse response to pemetrexed chemotherapy and survival outcomes. Although many previous studies have investigated *EXO1* polymorphism, especially rs1047840, in lung cancer, most of those studies evaluated the relationship between *EXO1* rs1047840 and the risk of lung cancer (33, 35, 44). Regarding the chemotherapy outcomes, one study by R.Li *et al*. showed the association between *EXO1* rs9350 and survival of patients receiving platinum-based chemotherapy in NSCLC (37). To the best of our knowledge, this is the first study to suggest a predictive role of *EXO1* rs1047840 in the therapeutic response to pemetrexed chemotherapy and also survival in lung adenocarcinoma patients. In addition, by the SNP selection using a database for miRNA binding site variants, we could suggest that the rs1047840-induced change in miRNA binding efficiency may be a mechanism of the association between *EXO1* rs1047840 and lung cancer. Alternatively, the *EXO1* rs1047840G>A is a non-synonymous SNP (E589K) with an amino acid change, which may affect EXO1 protein functions. Further studies are required to understand the role of EXO1 in the mechanism of pemetrexed resistance. On the other hand, potential oncogenic roles of EXO1 have also been suggested, which may be another possible mechanism of the association between *EXO1* rs1047840G>A and worse clinical outcomes after pemetrexed chemotherapy. Expression of degradation-resistant EXO1 could result in DNA hyper-resection in homologous recombination, which severely compromised DSBR and lead to chromosomal instability 43. A bioinformatic analysis study showed that EXO1 was identified as one of the hub genes which were markedly associated with poor prognosis in patients with lung cancer 44. Recently, Zhou *et al.* showed that *EXO1* was overexpressed in lung adenocarcinoma tissues and the high expression of *EXO1* was associated with poor prognosis 45. By bioinformatic analyses using public database, they revealed the correlation between increased *EXO1* expression and decreased tumor infiltrating B cells and CD4+ T cells, suggesting that EXO1 may induce an immune-suppressive tumor microenvironment in lung adenocarcinoma. Given that immune checkpoint blockade combined with pemetrexed-platinum doublet chemotherapy has been approved as the standard first-line treatment for lung adenocarcinoma, it will be worth to further evaluate *EXO1* rs1047840G>A as a biomarker to predict clinical outcomes of combined immunotherapy and pemetrexed chemotherapy.

In this study, *CAMKK2* rs1653586G>T was significantly related with the worse response to pemetrexed and survival outcome in lung adenocarcinoma patients. Calmodulin (CaM) is an intracellular calcium receptor which controls the downstream calcium/calmodulin kinase (CaMK) cascade that has been associated with various metabolic diseases 46. Calcium/calmodulin-dependent kinase kinase 2 (CAMKK2) belongs to the subfamily of calcium/calmodulin-dependent protein kinase and is involved in the maintenance of energy balance, adiposity, glucose homeostasis, inflammation, and cancer 47. *CAMKK2* is generally upregulated in several cancers, including prostate cancer, and high expression of *CAMKK2* was correlated with poor overall survival in hepatocellular carcinoma and glioma 48-50. It was reported that knockdown of *CAMKK2* potentiated the effect of carboplatin in ovarian cancer by modulating Akt pathway 51. However, the role of *CAMKK2* in the pathogenesis and prognosis of lung cancer has yet to be clarified. Further study is required to understand the molecular mechanism of the association between the genetic variants and clinical outcomes.

There are some limitations in this study. First, because pemetrexed maintenance regimen was not approved in the early part of the enrollment period, maintenance therapy was not prescribed for some patients who are considered eligible for the maintenance therapy by the current treatment guidelines. Although maintenance therapy was adjusted for in the multivariate analysis, the prognostic differences between patients with and without pemetrexed maintenance therapy could not be ignored. Second, as a single center retrospective study, the treatment other than pemetrexed in the course of treatment including targeted agents could not be strictly controlled in this study, which should be considered in the interpretation of the survival analysis. Third, the study cohort is relatively small. Although stratified analyses may show the clinical significance of *EXO1* rs1047840 and *CAMKK2* rs1653586 in different subgroups of patients, i.e. stage III/IV and postsurgical recurrence groups, this study is underpowered for stratification analyses or propensity score matching. Therefore, a well-designed and properly powered study is warranted to validate our findings.

In conclusion, two SNPs in miRNA binding sites, especially *EXO1* rs1047840G>A, were associated with the treatment response to pemetrexed chemotherapy and survival in lung adenocarcinoma patients. Evaluation of genetic variants in miRNA binding sites may be useful for refining therapeutic decisions in the treatment of lung adenocarcinoma by helping to identify subgroups of patients who will benefit from pemetrexed chemotherapy. To verify *EXO1* rs1047840G>A and *CAMKK2* rs1653586G>T as biomarkers for predicting clinical outcomes, the results of this study need to be further tested in a larger population with diverse ethnicity.

## Supplementary Material

Supplementary table.

## Figures and Tables

**Table 1:** Univariate analysis for response to chemotherapy and overall survival by clinical variables

	No. of	Response to chemotherapy				Overall survival				
		responders^a^	nonresponders^a^	OR (95% CI)	*P*	MST	95% CI	Log-Rank	HR (95% CI)	*P*
Variables	cases	(CR+PR)	(SD+PD)			(months)		*P*		
Overall	314	131 (41.7)^a^	183 (58.3)			24.1	21.7-25.9			
**Age (years)**										
≤ 65	177	65 (36.7)	112 (63.3)	1.00		25.8	23.6-31.1		1.00	
>65	137	66 (48.2)	71 (51.8)	1.60 (1.02-2.52)	0.04	21.1	17.2-25.1	0.08	1.26(0.97-1.63)	0.08
**Sex**										
Male	151	64 (42.4)	87 (57.6)	1.00		16.6	14.6-20.9		1.00	
Female	163	67 (41.1)	96 (58.9)	0.95 (0.61-1.49)	0.82	28.5	25.1-34.3	<0.0001	0.60(0.46-0.78)	0.0001
**Smoking status**										
Never	165	70 (42.4)	95 (57.6)	1.00		28.6	24.7-54.4		1.00	
Ever	149	61 (40.9)	88 (59.1)	0.94 (0.60-1.47)	0.79	16.4	14.5-22.5	0.0002	1.64(1.27-2.12)	0.0002
**Stage**										
III/IV	244	102 (41.8)	142 (58.2)	1.00		22.9	17.9-24.6		1.00	
Postsurgical recurrence	70	29 (41.4)	41 (58.6)	0.99 (0.57-1.69)	0.96	34.9	25.9-44.6	0.001	0.59(0.43-0.82)	0.001
**PS ECOG**										
0	102	45 (44.1)	57 (55.9)	1.00		29.5	24.6-35.4		1.00	
1-2	212	86 (40.6)	126 (59.4)	0.87 (0.54-1.39)	0.55	21.7	17.4-24.6	0.01	1.46(1.1-1.94)	0.01
**TKI benefit**										
No	199	95 (47.7)	104 (52.3)	1.00		15.2	14.0-17.2		1.00	
Yes	115	36 (31.3)	79 (68.7)	0.50 (0.31-0.81)	0.01	39.4	34.4-44.3	<0.0001	0.31(0.24-0.42)	<0.0001
**Maintenance^b^**										
No	113	52 (46.0)	61 (54.0)	1.00		14.3	10.8-17.1		1.00	
Yes	67	45 (67.2)	22 (32.8)	2.40 (1.28-4.50)	0.01	27.2	16.6-	<0.0001	0.41(0.27-0.62)	<0.0001
**Regimen**										
Pem/Cis	180	97 (53.9)	83 (46.1)	1.00		16.6	14.6-20.9		1.00	
Pem alone	134	34 (25.4)	100 (74.6)	0.29 (0.18-0.47)	<0.0001	28.8	25.4-35.4	0.0001	0.60(0.46-0.78)	0.0001

Abbreviation: CR, complete response; PR, partial response; SD, stable disease; PD, progressive disease; OR, odds ratio; MST, median survival time; CI, confidence interval; HR, hazard ratio; PS, performance status; ECOG, Eastern Cooperative Oncology Group; TKI, tyrosine kinase inhibitor; Pem, pemetrexed; Cis, cisplatin. ^a^ Row percentage. ^b^Among patients with Pem/Cis.

**Table 2:** Summary of 7 SNPs and response to chemotherapy

ID No.^a^	Target Gene	miRNA	Alleles	CR (%)	MAF	HWE-*p*	*P*^b^ for response		
							dominant	recessive	codominant
rs1047840	*EXO1*	hsa-miR-30e	GA	97.77	0.22	0.17	0.001	0.38	0.002
rs1653586	*CAMKK2*	hsa-miR-185	GT	99.68	0.09	0.08	0.002	-	0.002
rs2295865	*SUPT16H*	hsa-miR-186	CA	98.09	0.11	0.26	0.05	0.99	0.03
rs11541557	*ARF1*	hsa-miR-92a	GT	98.09	0.10	0.06	0.03	-	0.03
rs6698826	*RAB3B*	hsa-miR-27b	CA	95.54	0.14	0.72	0.01	0.85	0.01
rs3762158	*SUPT16H*	has-miR-484	GC	93.95	0.12	0.27	0.05	0.99	0.04
rs4705	*PDGFRL*	hsa-miR-25	CT	98.73	0.47	0.41	0.87	0.04	0.27

Abbreviation: CR, call rate; MAF, minor allele frequency; and HWE, Hardy-Weinberg equilibrium. ^a^Information about SNPs and SNP ID were obtained from NCBI database (https://www.ncbi.nlm.nih.gov/). The transcription start site was counted as +1 in reference sequences. ^b^*P* values were calculated by multivariate regression analysis, adjusted for age, sex, smoking status, stage, ECOG performance status, and chemotherapy regimen.

**Table 3:** Summary of 16 SNPs and overall survival

ID No.^a^	Target Gene	miRNA	Alleles	CR (%)	MAF	HWE-*p*	*P*^b^ for overall survival		
							dominant	recessive	codominant
rs1047840	*EXO1*	hsa-miR-30e	GA	97.77	0.22	0.17	0.04	0.62	0.07
rs1653586	*CAMKK2*	hsa-miR-185	GT	99.68	0.09	0.08	0.02		0.02
rs17445840	*CDH2*	hsa-miR-615-3p	CT	98.73	0.04	0.52	0.04	0.19	0.03
rs6934058	*CDC5L*	hsa-miR-505	TC	97.77	0.41	0.46	0.04	0.26	0.05
rs1056471	*HADHB*	hsa-miR-99a	GC	98.73	0.16	0.22	0.002	0.95	0.01
rs296888	*HNRNPK*	hsa-miR-615-3p	CT	99.36	0.28	0.71	0.01	0.30	0.01
rs3212986	*CD3EAP*	hsa-miR-92a	GT	98.09	0.27	0.24	0.02	0.45	0.03
rs12449580	*AIPL1*	has-miR-3615	CG	98.41	0.39	0.29	0.04	0.05	0.01
rs1318648	*ESPL1*	hsa-miR-149	TG	96.50	0.30	0.84	0.04	0.01	0.01
rs2076345	*TCEB3*	hsa-miR-320b	CT	93.63	0.25	0.17	0.04	0.76	0.10
rs2228128	*POLR2A*	has-miR-744	TC	93.63	0.06	0.28	0.05	0.97	0.02
rs3217933	*CCND2*	hsa-miR-17	TC	99.68	0.09	0.68	0.59	0.004	0.39
rs7654	*TPM3*	hsa-miR-615-3p	CA	99.68	0.08	0.15	0.62	0.01	0.35
rs2306409	*GTPBP4*	hsa-miR-16	TC	98.41	0.39	0.84	0.86	0.004	0.15
rs7091596	*PARD3*	hsa-miR-93*	AT	98.09	0.24	0.63	0.09	0.02	0.03
rs2261988	*UHRF1*	has-miR-615-3p	CA	98.41	0.15	0.16	0.09	0.06	0.04

Abbreviation: CR, call rate; MAF, minor allele frequency; and HWE, Hardy-Weinberg equilibrium. ^a^Information about SNPs and SNP ID were obtained from NCBI database (https://www.ncbi.nlm.nih.gov/). The transcription start site was counted as +1 in reference sequences. ^b^*P* values were calculated using multivariate Cox proportional hazard models, adjusted for age, sex, smoking status, stage, ECOG performance status, TKI benefit, maintenance therapy, and chemotherapy regimen.

**Table 4:** Associations between *EXO1* rs1047840 and *CAMKK2* rs1653586 and clinical outcomes

Polymorphism/Genotype	Target Gene	miRNA	No. of cases (%)^a^	Response				Overall Survival	
				Responders (%)^b^	Non-responders (%)^b^	OR (95% CI)^c^	*P* ^c^	HR (95% CI)^d^	*P* ^d^
rs1047840^f^	*EXO1*	hsa-miR-30e							
GG	(cds-non)		191 (62.2)	94 (49.2)	97 (50.8)	1.00		1.00	
GA			97 (31.6)	28 (28.9)	69 (71.1)	0.40 (0.23-0.69)	0.001	1.35 (1.01-1.81)	0.05
AA			19 (6.2)	6 (31.6)	13 (68.4)	0.47 (0.16-1.37)	0.17	1.28 (0.75-2.21)	0.37
Dominant						0.41 (0.24-0.68)	0.001	1.34 (1.01-1.77)	0.04
Recessive						0.63 (0.22-1.79)	0.38	1.14 (0.67-1.94)	0.62
*P_trend_* ^ e^						0.52 (0.34-0.79)	0.002	1.22 (0.98-1.51)	0.07
rs1653586^f^	*CAMKK2*	hsa-miR-185							
GG	(UTR-3)		257 (82.1)	118 (45.9)	139 (54.1)	1.00		1.00	
GT			56 (17.9)	12 (21.4)	44 (78.6)	0.33 (0.16-0.67)	0.002	1.50 (1.06-2.13)	0.02
TT			0 (0.0)	0 (0.0)	0 (0.0)	-	-	-	-
Dominant						0.33 (0.16-0.67)	0.002	1.50 (1.06-2.13)	0.02
Recessive						-	-	-	-
*P_trend_* ^ e^						0.33 (0.16-0.67)	0.002	1.50 (1.06-2.13)	0.02

Abbreviations: OR, odds ratio; CI, confidence interval; HR, hazard ratio. ^a^Column percentage. ^b^Row percentage. ^c^OR, 95% CI, and their corresponding *P* values were calculated by multivariate regression analysis, adjusted for age, sex, smoking status, stage, ECOG performance status, and chemotherapy regimen. ^d^HRs, 95% CIs and their corresponding *P* values were calculated using multivariate Cox proportional hazard model, adjusted for age, sex, smoking status, stage, ECOG performance status, TKI benefit, maintenance therapy, and chemotherapy regimen. ^e^*P_trend_* for the additive model. ^f^Genotype failure: 7 cases for rs1047840, 1 for rs1653586.
