# Supplementary material for: Exonuclease 1 genetic variant is associated with clinical outcomes of pemetrexed chemotherapy in lung adenocarcinoma
Source: J Cancer. 2022 Dec 7;13(15):3701–9. doi: 10.7150/jca.78498 (PMC9809306; doi:10.7150/jca.78498)
Supplement: Supplementary file 1 — Supplementary table. [file jcav13p3701s1.pdf]

Supplementary Table 1. Summary of 74 SNPs and chemotherapy response and survival.

SNP ID[a]	Target Gene	miRNA	Alleles	CR (%)	MAF	HWE-*P*	*P* for response[b]			*P* for overall survival[c]		
							Dominant	Recessive	Codominant	Dominant	Recessive	Codominant
rs1047840	*EXO1*	hsa-miR-30e	GA	97.77	0.22	0.17	0.001	0.38	0.002	0.04	0.62	0.07
rs1653586	*CAMKK2*	hsa-miR-185	GT	99.68	0.09	0.08	0.002		0.002	0.02		0.02
rs2295865	*SUPT16H*	hsa-miR-186	CA	98.09	0.11	0.26	0.05	0.99	0.03	0.38	0.56	0.34
rs11541557	*ARF1*	hsa-miR-92a	GT	98.09	0.10	0.06	0.03		0.03	0.46		0.46
rs6698826	*RAB3B*	hsa-miR-27b	CA	95.54	0.14	0.72	0.01	0.85	0.01	0.09	0.23	0.19
rs3762158	*SUPT16H*	has-miR-484	GC	93.95	0.12	0.27	0.05	0.99	0.04	0.43	0.58	0.39
rs4705	*PDGFRL*	hsa-miR-25	CT	98.73	0.47	0.41	0.87	0.04	0.27	0.69	0.73	0.64
rs17445840	*CDH2*	hsa-miR-615-3p	CT	98.73	0.04	0.52	0.26	0.99	0.23	0.04	0.19	0.03
rs6934058	*CDC5L*	hsa-miR-505	TC	97.77	0.41	0.46	0.26	0.80	0.53	0.04	0.26	0.05
rs1056471	*HADHB*	hsa-miR-99a	GC	98.73	0.16	0.22	0.23	0.54	0.20	0.002	0.95	0.01
rs296888	*HNRNPK*	hsa-miR-615-3p	CT	99.36	0.28	0.71	0.09	0.76	0.14	0.01	0.30	0.01
rs3212986	*CD3EAP*	hsa-miR-92a	GT	98.09	0.27	0.24	0.59	0.90	0.64	0.02	0.45	0.03
rs12449580	*AIPL1*	has-miR-3615	CG	98.41	0.39	0.29	0.82	0.49	0.84	0.04	0.05	0.01
rs1318648	*ESPL1*	hsa-miR-149	TG	96.50	0.30	0.84	0.15	0.89	0.28	0.04	0.01	0.01
rs2076345	*TCEB3*	hsa-miR-320b	CT	93.63	0.25	0.17	0.46	0.41	0.35	0.04	0.76	0.10
rs2228128	*POLR2A*	has-miR-744	TC	93.63	0.06	0.28	0.93	0.41	0.79	0.05	0.97	0.02
rs3217933	*CCND2*	hsa-miR-17	TC	99.68	0.09	0.68	0.28	0.99	0.43	0.59	0.004	0.39
rs7654	*TPM3*	hsa-miR-615-3p	CA	99.68	0.08	0.15	0.21	0.52	0.35	0.62	0.01	0.35
rs2306409	*GTPBP4*	hsa-miR-16	TC	98.41	0.39	0.84	0.30	0.87	0.51	0.86	0.004	0.15
rs7091596	*PARD3*	hsa-miR-93*	AT	98.09	0.24	0.63	0.58	0.21	0.98	0.09	0.02	0.03
rs2261988	*UHRF1*	has-miR-615-3p	CA	98.41	0.15	0.16	0.71	0.70	0.86	0.09	0.06	0.04
rs2290890	*SYK*	hsa-miR-99b*	GA	98.09	0.22	0.35	0.93	0.87	0.99	0.19	0.12	0.10
rs9523	*NUP62*	hsa-miR-1914	AG	98.09	0.43	0.07	0.31	0.69	0.62	0.47	0.59	0.42
rs20554	*EP300*	hsa-miR-23b	GA	98.73	0.16	0.46	0.33	0.94	0.39	0.09	0.28	0.06
rs40311	*GSPT1*	hsa-miR-183	GC	99.04	0.17	0.15	0.52	0.52	0.44	0.07	0.44	0.07
rs3786362	*TYMS*	hsa-miR-615-3p	TC	93.63	0.15	0.51	0.21	0.64	0.21	0.95	0.30	0.77
rs7195830	*CYBA*	hsa-miR-320a	GA	97.77	0.21	0.38	0.38	0.90	0.48	0.46	0.33	0.79
rs1049910	*LMNB2*	hsa-miR-193b	CG	99.04	0.31	0.60	0.38	0.71	0.61	0.09	0.18	0.06
rs157705	*MAP3K7*	hsa-miR-1226*	GA	97.45	0.33	0.26	0.15	0.14	0.08	0.65	0.15	0.77
rs16830621	*MGAT5*	hsa-miR-769-5p	GA	97.45	0.03	0.58	0.57		0.57	0.81		0.81
rs2297441	*RTEL1*	hsa-miR-615-3p	GA	97.13	0.28	0.91	0.27	0.26	0.70	0.19	0.34	0.15
rs2589668	*MARCH6*	hsa-let-7b	CT	97.45	0.26	0.65	0.78	0.68	0.69	0.59	0.61	0.84
rs3826810	*LDLR*	hsa-miR-92a	GA	97.45	0.09	0.61	0.17	0.58	0.27	0.36	0.45	0.46
rs4919551	*BTRC*	hsa-let-7e	CG	98.73	0.10	1.00	0.50	0.99	0.40	0.63	0.59	0.75
rs6573	*RAP1A*	hsa-let-7e	CA	96.50	0.05	0.65	0.37	0.99	0.34	0.75	0.57	0.65
rs1013062	*DEK*	hsa-miR-96	CT	98.09	0.31	0.71	0.89	0.64	0.92	0.56	0.49	0.45
rs1140034	*ADCK2*	has-let-7b	TC	98.73	0.07	0.19	0.38		0.38	0.39		0.39
rs11644	*TTL*	hsa-miR-92a	AG	99.04	0.41	0.00	0.21	0.99	0.24	0.14	0.80	0.14
rs14235	*BCKDK*	hsa-miR-92b	AG	98.73	0.07	0.19	0.07		0.07	0.41		0.41
rs16855615	*CCNT2*	hsa-miR-484	GA	98.73	0.27	0.95	0.70	0.09	0.31	0.63	0.68	0.58
rs1965024	*SALL1*	hsa-miR-423-5p	TC	98.41	0.32	0.80	1.00	0.49	0.75	0.33	0.89	0.53

rs2230724	*JAK2*	hsa-miR-16	GA	99.36	0.40	0.62	0.30	0.94	0.45	0.15	0.36	0.14
rs2230738	*ADCY9*	hsa-miR-877*	AG	98.73	0.35	0.08	0.29	0.14	0.99	0.51	0.74	0.53
rs3182911	*COPS3*	hsa-miR-484	TC	98.73	0.40	0.32	0.16	0.29	0.66	0.85	0.39	0.56
rs3203733	*MTUS1*	hsa-miR-1296	AG	98.73	0.23	0.81	0.76	0.26	0.50	0.31	0.80	0.35
rs3814026	*ANAPC1*	hsa-miR-744	TC	98.73	0.48	0.84	0.16	0.28	0.13	0.60	0.09	0.18
rs4074826	*HIPK2*	hsa-miR-423-5p	CT	98.73	0.17	0.72	0.52	0.38	0.40	0.55	0.78	0.54
rs461155	*ETS2*	hsa-miR-149	AG	98.41	0.46	0.60	0.91	0.81	0.83	0.20	0.41	0.74
rs5744857	*POLE*	hsa-miR-92a	AG	98.73	0.32	0.55	0.56	0.67	0.52	0.38	0.16	0.93
rs7116130	*PSMD13*	hsa-miR-93*	AG	98.41	0.22	0.07	0.23	0.29	0.17	0.52	0.73	0.51
rs8444	*LASS2*	hsa-miR-20a	CT	98.09	0.40	0.59	0.66	0.76	0.89	0.79	1.00	0.85
rs1049434	*SLC16A1*	hsa-miR-615-3p	AT	99.04	0.36	1.00	0.79	0.18	0.63	0.06	0.42	0.07
rs1051709	*PTK7*	hsa-miR-193b	TC	98.73	0.40	0.51	0.96	0.29	0.60	0.51	0.43	0.39
rs1061157	*BMPR2*	hsa-miR-93*	GA	99.04	0.06	0.97	0.21	0.99	0.19	0.66	0.93	0.69
rs1111667	*ERO1LB*	hsa-miR-106b*	AG	99.04	0.29	0.24	0.47	0.46	0.38	0.86	0.51	0.87
rs1707303	*PIK3R3*	hsa-miR-346	GT	99.04	0.30	0.22	0.20	0.79	0.25	0.46	0.52	0.39
rs1803621	*GAPDH*	hsa-miR-149	CT	98.41	0.50	0.00	0.48	0.81	0.51	0.82	0.63	0.87
rs1805329	*RAD23B*	hsa-miR-92a	CT	98.09	0.19	0.15	0.64	0.46	0.51	0.94	0.70	0.84
rs2288539	*NR2F6*	has-miR-196a	CT	99.36	0.19	0.45	0.55	0.78	0.55	0.80	0.53	0.99
rs4485435	*FASN*	hsa-miR-326	GC	98.41	0.15	0.95	0.05	0.67	0.07	0.78	0.98	0.82
rs1043805	*FKBP5*	hsa-miR-93*	TA	98.09	0.18	0.33	0.98	0.92	0.95	0.16	0.97	0.21
rs10467153	*DYRK2*	hsa-miR-378	TC	96.18	0.39	0.99	0.12	0.20	0.08	0.85	0.32	0.72
rs11196251	*TCF7L2*	hsa-miR-324-5p	CT	97.13	0.25	0.05	0.35	0.17	0.19	0.91	0.06	0.53
rs2229534	*ACADS*	hsa-miR-92a	GA	99.36	0.21	0.93	0.61	0.17	0.35	0.71	0.45	0.95
rs2854464	*ACVR1B*	hsa-miR-182	AG	97.77	0.39	0.40	0.76	0.68	0.67	0.89	0.67	0.90
rs3088440	*CDKN2A*	hsa-miR-10b	GA	97.77	0.10	0.18	0.38	0.99	0.33	0.41	0.98	0.44
rs6089219	*CABLES2*	has-miR-106b*	TG	97.77	0.31	0.09	0.49	0.83	0.68	0.18	0.90	0.31
rs7081076	*SORBS1*	hsa-miR-320a	CA	98.41	0.09	0.85	0.74	0.99	0.52	0.26	0.80	0.33
rs989902	*PTPN13*	hsa-miR-186	AC	96.18	0.40	0.65	0.28	0.65	0.32	0.34	0.73	0.64
rs1569238	*REPS1*	hsa-miR-193b	GA	93.63	0.18	0.26	0.84	0.80	0.80	0.29	0.16	0.52
rs2074216	*DVL2*	has-miR-484	GA	93.63	0.36	0.64	0.17	0.89	0.34	0.57	0.67	0.53
rs2269529	*MYH9*	hsa-miR-1296	CT	93.63	0.41	0.41	0.44	0.96	0.57	0.61	0.47	0.98
rs2279130	*ARRB1*	hsa-miR-92a	CT	93.31	0.23	0.61	0.98	0.33	0.70	0.26	0.77	0.42
rs9479	*PML*	hsa-miR-378	GA	93.63	0.32	0.09	0.18	0.99	0.30	0.85	0.16	0.48

Abbreviation: CR, call rate; MAF, minor allele frequency; HWE, Hardy-Weinberg equilibrium.

[a]Information about SNPs and SNP ID were obtained from NCBI database (http://ncbi.nih.gov). The transcription start site was counted as +1 in reference sequences.

[b]*P* values were calculated by multivariate regression analysis, adjusted for age, sex, smoking status, stage, ECOG performance status, and chemotherapy regimen.

[c]*P* values were calculated using multivariate Cox proportional hazard models, adjusted for age, sex, smoking status, stage, ECOG performance status, TKI benefit, maintenance therapy, and chemotherapy regimen.
